# Data in support of the detection of genetically modified organisms (GMOs) in food and feed samples

**DOI:** 10.1016/j.dib.2016.02.035

**Published:** 2016-02-20

**Authors:** Noor Alasaad, Hussein Alzubi, Ahmad Abdul Kader

**Affiliations:** General Commission for Scientific Agricultural Research (GCSAR), Biotechnology Department, Damascus, P.O. Box 12573, Syria

## Abstract

Food and feed samples were randomly collected from different sources, including local and imported materials from the Syrian local market. These included maize, barley, soybean, fresh food samples and raw material. GMO detection was conducted by PCR and nested PCR-based techniques using specific primers for the most used foreign DNA commonly used in genetic transformation procedures, *i.e*., *35S* promoter, T-*nos*, *epsps*, *cryIA(b)* gene and *nptII* gene.

The results revealed for the first time in Syria the presence of GM foods and feeds with glyphosate-resistant trait of *P35S* promoter and *NOS* terminator in the imported soybean samples with high frequency (5 out of the 6 imported soybean samples). While, tests showed negative results for the local samples. Also, tests revealed existence of GMOs in two imported maize samples detecting the presence of 35S promoter and *nos* terminator. Nested PCR results using two sets of primers confirmed our data.

The methods applied in the brief data are based on DNA analysis by Polymerase Chain Reaction (PCR). This technique is specific, practical, reproducible and sensitive enough to detect up to 0.1% GMO in food and/or feedstuffs. Furthermore, all of the techniques mentioned are economic and can be applied in Syria and other developing countries. For all these reasons, the DNA-based analysis methods were chosen and preferred over protein-based analysis.

## Graphical abstract

**Figure:**
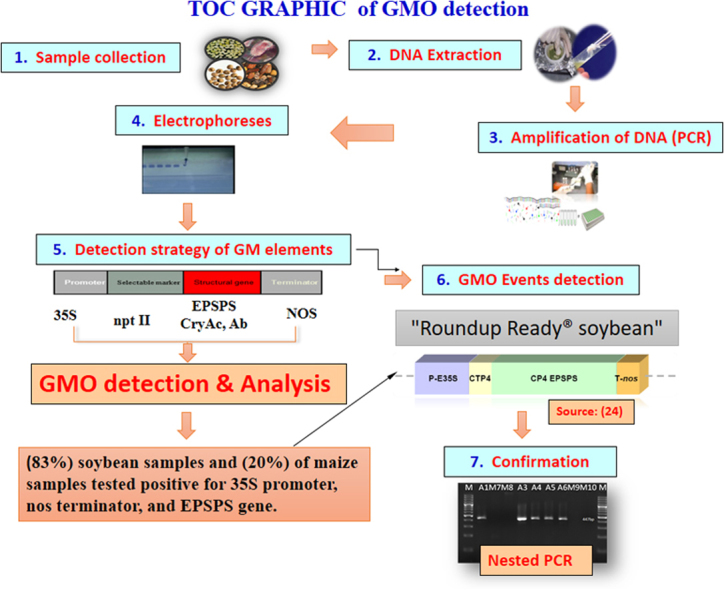

## Specifications Table

**Table:** 

Subject area	*Biology, Biotechnology*
More specific subject area	*Genetics and molecular biology, plant biotechnology, Genetically Modified Organisms (GMOs)*
Type of data	*Table, text file, primer sequences and characteristics, figures*
How data was acquired	*Polymerase Chain Reaction (PCR) Thermocycler System, (Mastercycler Eppendrof) Germany.*
*Agarose Gel Electrophoresis chamber tray system (Bio Rad, Germany), Power supply (PAC 300, Germany).*
*Gel documentation system (LABORTECHNIK TCP26 M) (EEC)*
Data format	*Raw, analyzed*
Experimental factors	*DNA extraction and optimization. Primers optimization, Optimization of PCR conditions.*
Experimental features	*Genomic DNA was extracted from the different samples tested using CTAB method for seed samples and from leaves by the modified CTAB method of Doyle and Doyle (1990), while by alkaline lysis method for the positive samples.*
*Optimization of DNA extraction from fresh material and from seeds.*
*Different annealing temperatures were applied in programing the PCR according to the target gene and optimization experiment and based on literatures.*
Data source location	*GCSAR, Biotechnology department, Damascus, Syria*
Data accessibility	*Data are available within this article*

## Value of the data

• Insight about the market situation of imported products in comparison with the local products.
• This GMO testing data presented is considered the first and the pioneering data from Syria and support GMO detection in Syria and other neighboring countries for future investigation.
• Data presented shed light on the importance and methodologies of the GMO testing and its application.
• Screen and GMOs detection methodologies applied can be a keystone for risk assessment and evaluation of any food and feed products introduced into the market either as human food or as animal feed for biosafety-related investigation.
• Data of GMO detection is a critical and a prerequisite for enforcement of the biosafety law related to biotechnologically derived products.
• The data are based on DNA analysis by Polymerase Chain Reaction (PCR). This technique is specific, practical, reproducible and sensitive enough to detect up to 0.1% GMO in food and/or feedstuffs.
• Furthermore, all of the techniques mentioned are economic and can be applied in Syria and other developing countries.

## 1 Data

Foodstuff, feed and agricultural samples were randomly collected from different sources, including local and imported materials from the Syrian local market to screen them for detecting the presence of GMOs using PCR, nested PCR- and multiplex PCR-based techniques using specific primers for the most commonly used foreign DNA commonly used in genetic transformation procedures, *i.e*., 35S promoter, T-*nos*, *epsps*, *cryIA(b)* gene and *nptII* gene [1], [2], [3], [4], [5], [6], [7], [8], [9], [10], [11], [12], [13], [14], [15], [16], [17].

## 2 Experimental design, materials and methods

### 2.1 Sample collection

Thirty seven samples were randomly collected from different sources, including local and imported materials from the Syrian local market and can be categorized as the following:

– Maize (labeled as M1–M16), barley (B1–B2) and soybean (labeled as A1–A6) which are used mainly as animals feed.
– Fresh food samples: tomato (T1–T5), cucumber (C1–C3) used directly for human consumption.
– Raw material: soybean seeds, sunflower seeds (S1–S2), popcorn seeds (P1), rice (R) which will be used for processing oil and other applications.
– Several plasmids (PGIIMH35-2PS, TOP10 PGII35S CRYA(b), pBI-121, TOP10 PGII35S CRYA(c)) were used as a positive control.

### 2.2 DNA extraction and quantification

– CTAB (cetyltrimethyl ammonium bromide) extraction, the basis for the official German method, proposed by the CEN (European Committee for Standardization) in 2002 for the detection of genetically modified foods was used to extract genomic DNA from seed sample (maize soybean sunflower seeds, popcorn seeds, rice and barley).
– Genomic DNA was isolated from leaves of tomato and cucumber samples tested by the modified CTAB (hexadecyltrimethyl ammonium bromide) method of Doyle and Doyle [18].

For each sample, 0.5 g of grounded leaf tissue (grounded in an electric grinder) from a bulk of 10 plants was suspended in 2 ml of extraction buffer.

The suspension was mixed well, incubated at 60 °C for 30 min, followed by chloroform-isoamyl alcohol (24:1) extraction, and precipitation with 0.67 vol. isopropanol at −20 °C. The pellet formed after centrifugation at low speed for 5 min is then washed with 76% (v/v) ethanol and 10 mM NH4OAc. Then the DNA is suspended in TE buffer.

– Plasmid DNA from positive control samples was extracted by alkaline lysis as described by Sambrook et al. [19].

The quality and quantity of DNA extracted from samples were determined using spectrophotometer at 260 nm (A260) and 280 nm (A280) absorbance. The DNA purity was determined based on A260/A280 ratio.

### 2.3 PCR amplification

PCR amplification was carried out in a PCR mix of 25 µl. The final concentrations of each PCR were as follows: l× of 10× PCR buffer (fermentase); 100 ng of genomic DNA; 0.4 pm of each primers; 0.32 mM of dNTPs mix; 2 mM MgCl_2_; 0.5 unit/reaction of (Fermentas) Taq DNA polymerase.

**Oligonucleotide Primers** used in this study are listed in Table 1 [20]. All primers were synthesized by Eurofins MWG GmbH (Germany) and obtained in a lyophilized state. All primers were dissolved in TE buffer before use to obtain final concentration of 10 pmol/μl.

**For nested PCR**, the first reaction was done using the primer pair GMO9/GMO7 then followed by taking 1 µl from the PCR product as a template to make the second reaction with the primer pair GMO7/GMO8 and the master mix was the same as mentioned before.

**PCR program** used was as the following: initial denaturation (94 °C for 5 min); denaturation (94 °C for 1 min) then the annealing temperature changed according to each primer for 1 min, the extension was at 72 °C for 1 min; the number of cycles was 35 and the final extension was at 72 °C.

### 2.4 Agarose gel electrophoresis and documentation

Amplicons were analyzed in 2% agarose gel electrophoreses in a 1× TBE [10 mM Tris-base (pH 8); 2.75 g Boric acid/L; 1 mM EDTA (pH 8)] and visualized under UV transilluminator using SYBR® Safe DNA gel stain which exhibited very low mutagenicity compared to ethidium bromide, and it is not classified as hazardous waste or as a pollutant under U.S. federal regulations [21].

### 2.5 Data analysis

Data obtained were analyzed and interpreted.

## 3 Results

### 3.1 DNA extraction and amplification

Most of the DNA extracted by CTAB methods in this study showed a high molecular weight and high purity with A260/A280 ratios ranging from 1.8 to 2.0. The purity of DNA extracted from samples was confirmed by PCR amplification using soybean-specific (*lectin* gene) and maize-specific (*zein* gene) primers for samples derived from soybean and maize, respectively. These tests also indicated whether the other tested samples contained either soybean or maize materials, and if there was any contamination between DNA samples tested (15). The primer pair GM03/GM04 with amplicon size of (118 bp) is specific for the single copy of *lectin* gene LE1was used. On the other hand, the primer pair zein3/zein4 was used which is specific for the native maize *zein* gene (Ze1, coding for a 10 kD protein) and yields a PCR product of 277 bp size (13).

**Fig. 1:**
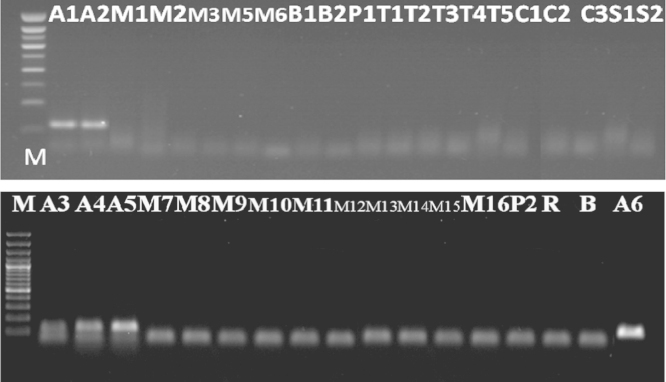
Detection of *lectin* gene in soybean samples tested (A1, A2, A3, A4, A5, and A6) (Results of PCR products of primer pair (GM03/GM04)) M: 100 bp DNA marker, B: negative control, lanes A1–A6: tested samples.

Using GM03/GM04 primers, all tested soybean samples gave positive results, while the result was negative in the other samples tested (Fig. 1). These results revealed that the DNA was successfully isolated, and the isolated DNA could be amplified with PCR using this specific primer without inhibition, where there was no contamination between DNA samples tested.

**Fig. 2:**
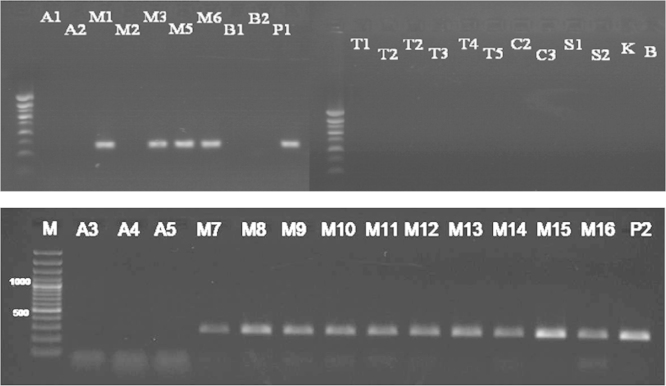
Detection of *zein* gene in maize samples tested (M1, M2, M3, M5, M6, M7, M8, M9, M10, M11, M12, M13, M14, M15, and M16) (Results of PCR products of primer pair zein3/zein4), M: 100 bp DNA marker, B: negative control, lanes A1–P2: tested samples.

Using zein3/zein4 primer, all tested Maize samples gave positive results. However, the result was negative in the other samples tested (Fig. 2). These results revealed that the DNA was successfully isolated, and the isolated DNA could be amplified with PCR without inhibition, where there was no contamination between DNA samples tested.

### 3.2 Screening method

Screening methods using the 35S promoter and NOS terminator sequences evidently are the most favorable candidates for broad method applicability (16). Most of the currently available GMOs worldwide contain any of three genetic elements: the cauliflower mosaic virus (CaMV) 35S promoter, the nonpalin synthase (nos) terminator or the kanamycin-resistance marker gene (*npt*II) for instance (3).

#### 3.2.1 Screening for the P35S promoter

**Fig. 3:**
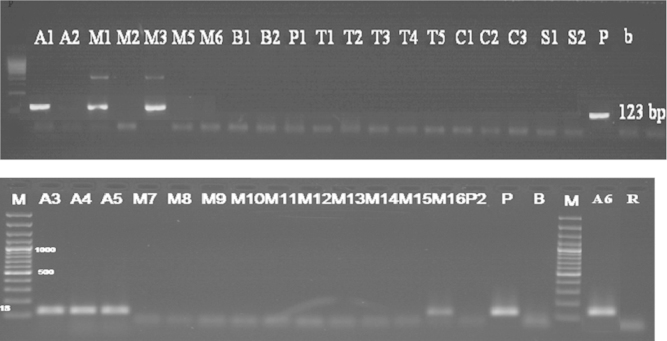
Detection of 35S promoter in samples (Results of PCR products of primer pairs p35S-cf3 and p35S-cf4), M: 100 bp DNA marker, B: negative control, P: positive control plasmid (PGIIMH35-2PS), lanes A1-R: tested samples.

After PCR amplifications of the *lectin* and *zein* genes, all the DNA stocks were subjected to PCR amplification of the 35S promoter which are specific to the 35S promoter originating from CaMV virus (22). The primer pair p35S-cf3 and p35S-cf4 (6) was used to detect one copy of this promoter. By using this primer pairs, visible band at 123 bp was found in the positive control (P), the soybean samples (A1, A3, A4, A5, A6) and the Maize samples (M1, M3, M16) (Fig. 3), which means that these soybean and maize samples are genetically modified containing this promoter. Whereas, no band at the expected size was shown in the other samples tested, which means that these samples do not contain that promoter.

**The plasmid (PGIIMH35-2PS)** was used as a positive control to be sure that the PCR condition was suitable for these primers used where the plasmid gave the same size of the expected fragment.

#### 3.2.2 Screening for the nos terminator

**Fig. 4:**
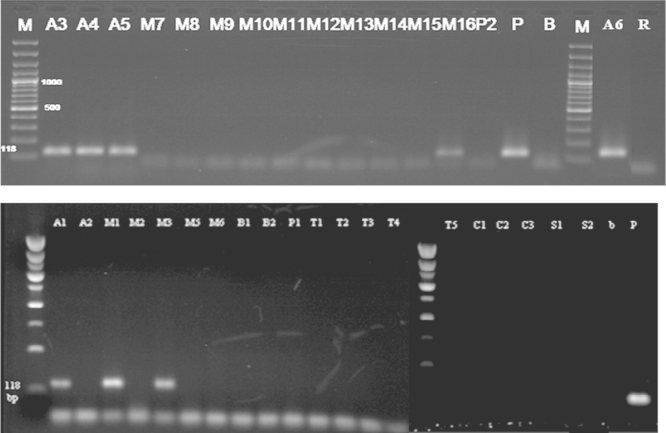
Detection of *nos* terminator in samples (Results of PCR products of primer pairs HA-nos118-f/HA-nos118-r), M: 100 bp DNA marker, B: negative control, P: positive control plasmid (PGIIMH35-2PS), lanes A1-R: tested samples.

The primer pairs HA-nos118-f and HA-nos118-r (6) was used to detect this terminator, where a visible band at 118 bp was found in the positive control (P), in Soybean samples tested (A1, A3, A4, A5, A6) and in Maize samples tested (M1, M3, M16) (Fig. 4), which means that these samples are genetically modified, whereas no visible band at the expected size was shown in the other samples, which means that these samples are not genetically modified using this terminator.

#### 3.2.3 Screening for glyphosate-tolerant trait by the presence of EPSPS gene

After the initial screening steps, specific detection was done to determine the structural genes of the introduced traits. Two main traits of interest mostly used in the construction of transgenic plants are herbicide tolerance and insect resistance. Herbicide tolerance is the leading trait in commercialized GM plants with 23 lines having been approved for cuor food and/or food and feed use worldwide [14], [15], [23].

**Fig. 5:**
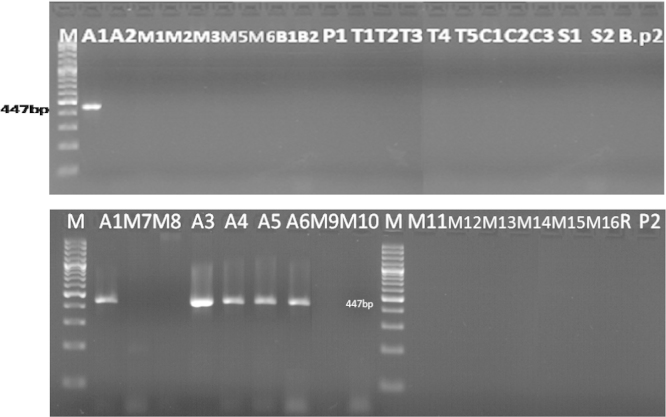
Detection of *epsps* gene in samples (Results of PCR products of primer pairs: GMO5, GMO9) M: 100 bp DNA marker, B: negative control, lanes A1–P2: Tested samples.

To detect this gene, the primers GMO5 and GMO9 (20) were used, where a visible band at 447 bp was detected in the soybean samples tested (A1, A3, A4, A5, A6) (Fig. 5), which means that these samples are genetically modified with this gene, while, no visible band at the expected size was detected in other samples tested, which means that these samples are not modified with this gene.

#### 3.2.4 Detection of *npt*II gene

The most frequently used transgene is *nptII*, originating from the *Escherichia coli* transposon 5. This gene confers resistance to Kanamycin [22].

**Fig. 6:**
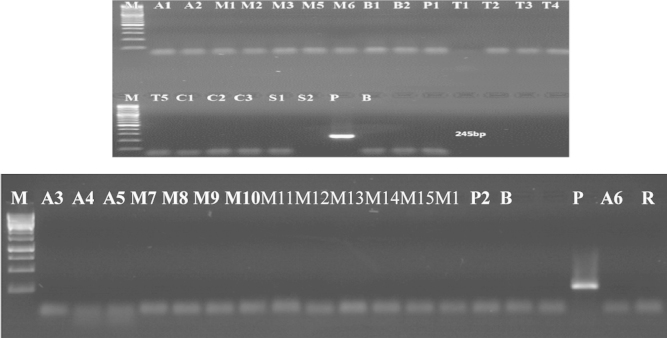
Detection of *nptII* gene in samples (Results of PCR products of primer pairs (nptIIf-nptIIr)) M: 100 bp DNA marker, B: negative control, P: positive control plasmid (pBI-121), lanes A1–P2: tested samples.

To detect this gene (nptIIf-nptIIr) primers were used where visible band at 254 bp was found just in the positive control plasmid (pBI-121) (Fig. 6), which means that these samples are not genetically modified with this selectable marker gene.

#### 3.2.5 Detection of *Cry* gene

There are several strains of Bt (*Bacillus thuringiensis*), each with differing Cry proteins. Scientists have identified more than 170 Cry proteins (9). Most of the Bt maize hybrids, targeted against European Corn Borer (*Ostrinia nubilalis*), produce only the Cry1A(b) protein (Bt176 maize line) (2).

The *cry1Ab* delta-endotoxin gene codes for the production of a naturally occurring insecticidal protein (derived from *B. thuringiensis* ssp. *kurstaki*). This gene was modified to optimize and maximize the expression of the Q-endotoxin CRYIA(b) protein in plants (9). 11bt1-11bt2 primers were used to detect this gene, which yields a PCR product of 200 bp where a visible band at 200 bp was detected only in the positive control plasmid (TOP10 PGII35S CRYA(b)), which means that these samples are not genetically modified with this structural gene.

In addition, Cry1Ac delta-endotoxin, derived from *B. thuringiensis* subp*.* kurstaki strain HD-73, encode resistance to European Corn Borer (ECB), a major insect pest of maize.

The primer pairs Cry1Ac 699–Cry1Ac 1440 was used to detect this gene, which yields a PCR product of 742 bp, while the plasmid (TOP10 PGII35SCRYA(c)) was used as a positive control.

The primers gave positive result only in the positive control plasmid, while the negative results were demonstrated in the other samples tested, which means that there is no modification with this gene.

### 3.3 Confirmation of results

#### Nested PCR

Confirmation/verification of the identity of the amplicon is necessary to assure that the amplified DNA is really corresponding to the chosen target sequence and is not a by-product of unspecific binding of the primers. Several methods are available for this purpose, first by using gel electrophoresis, but there is risk of artifact having the same size of the target sequence may have been amplified. Therefore, the PCR products should additionally be verified for their restriction endonuclease profile (8). The second method could be used is to subject the PCR product to second round of PCR cycle in a technique that is called nested PCR (5, 7). Here, two different sets of primers – an outer and an inner (=nested) pair – are being used within the target region in two consecutive rounds of PCR amplifications. This strategy reduces substantially the problem of un-specific amplification, as the probability for the inner pair of primers of finding complementary sequences within the non-specific amplification products of the outer pair is extremely low.

**Fig. 7:**
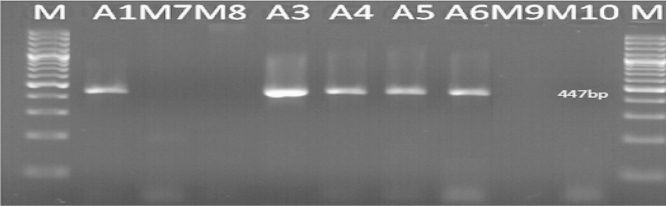
Detection of *epsps* gene in some samples tested (Results of PCR products of primer pairs (GMO9/GMO5)) M: 100 bp DNA marker, B: negative control, lanes A1–M10: tested samples.

The primer pairs GMO9/GMO5 and GMO8/GMO7 were designed for the transgene of roundup ready soybean by nested PCR, the external primer GMO9/GMO5 are complementary to the *cp4 epsps* gene/CaMV 35S promoter, the amplification of DNA with this primer resulted in an amplicon of 447 bp (Fig. 7).

**Fig. 8:**
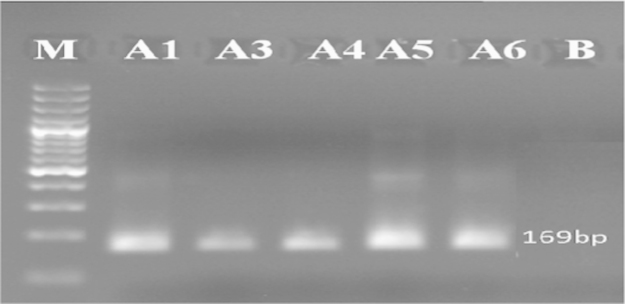
Detection of *epsps* gene in some samples tested by nested PCR (Results of PCR products of primer pairs (GMO8/GMO7)) M: 100 bp DNA marker, B: negative control, lanes A1–A6: tested samples.

The internal primers GMO8/GMO7, are complementary to the *epsps petunia* gene and to the CaMV 35S promoter. The amplification of DNA with these internal primer resulted in a fragment of 169 bp (Fig. 8).

This result confirms that the amplified DNA is really corresponding to *epsps* gene and is not a by-product of unspecific binding of the primers.

**Fig. 9:**
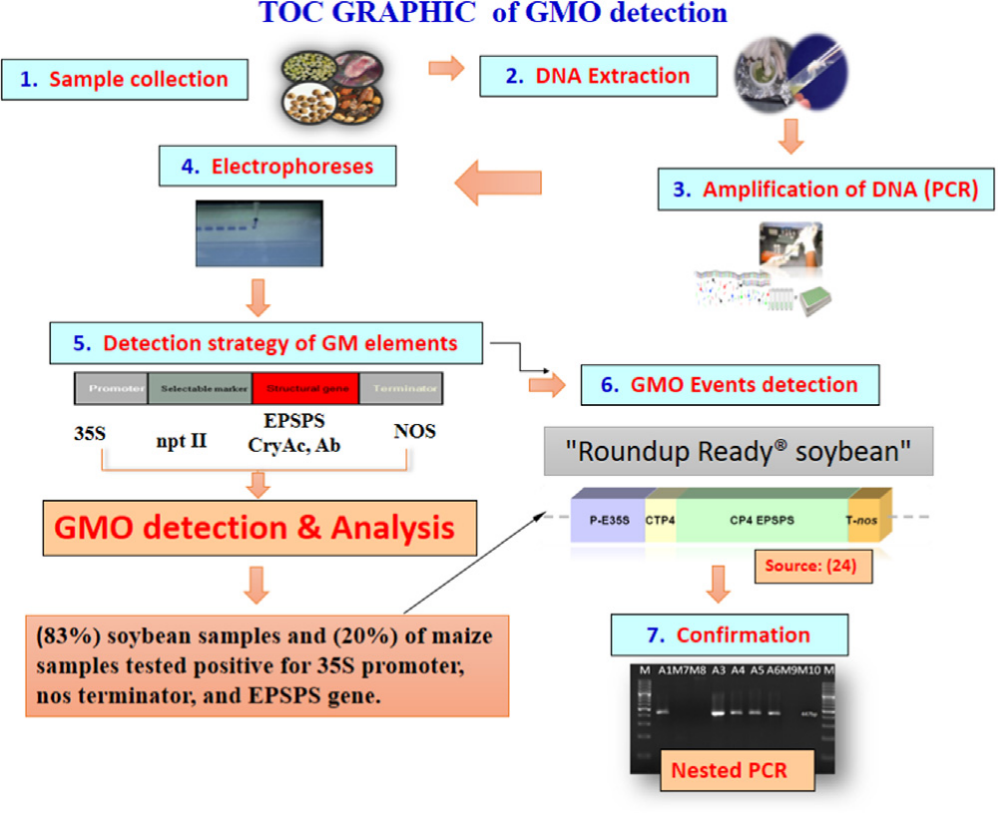
Summary of the methodologies and data presented [24].

Fig. 9 summarizes the methodologies and data presented in this brief data.

## Figures and Tables

**Table 1:** Oligonucleotide primer pairs sequences used and their target element.

**No.**	**Primer**	**Sequences**	**Target gene**	**Amplicon size (bp)**	**Annealing temperature**
1	GMO3	GCCCTCTACTCCACCCCCATCC	Lactin gene	118	63
GMO4	GCCCATCTGCAAGCCTTTTTGTG
2	ZEIN3	AGTGCGACCCATATTCCAG	Zein gene	277	60
ZEIN4	GACATTGTGGCATCATCATTT
3	P35s-cf3	CCACGTCTTCAAAGCAAGTGG	35S promoter	123	60
P35s-cf4	TCCTCTCCAAATGAAATGAACTTCC
4	HA-NOS118F	GCATGACGTTATTTATGAGATGGG	Nos terminator	118	62
HA-NOS118R	GACACCGCGCGCGATAATTTATCC
5	Cry1Ac 699	GTTCAGGAGAGAATTGACCC	Cry1Ac	742	60
Cry1Ac 1440	CTTCACTGCAGGGATTTGAG
6	Npt II F	CGCAGGTTCTCCGGCCGCTTGGGTGG	nptII	254	59
Npt II R	GCAGCCAGTCCCTTCCCGCTTCAG
7	11BT1	CAGGCAAGGATTCTCCCACA	CRYIA(b)	200	60
11BT2	CGACAGAAGTTCCAGATCCA
8	GMO5	CCACTGACGTAAGGGATGACG	EPSPS gene	447	60
GMO9	CATGAAGGACCGGTGGGAGAT
9	GMO7	ATCCCACTATCCTTCGCAAGA	EPSPS gene	169	54
GMO8	TGGGGTTTATGGAAATTGGAA
