# Corrigendum to 'Data in support of the detection of genetically modified organisms (GMOs) in food and feed samples' [Data Brief 7 (2016) pp. 243–252]

**DOI:** 10.1016/j.dib.2016.09.004

**Published:** 2016-09-30

Concern has been raised about the similarities between the lower panel of Fig. 3 and the upper panel of Fig. 4 in the above article. To avoid any misunderstanding, the authors would like to replace Fig. 3 by the replacement Fig. 3 below. Fig. 4 is unchanged and is also reproduced below.

**Fig. 3:**
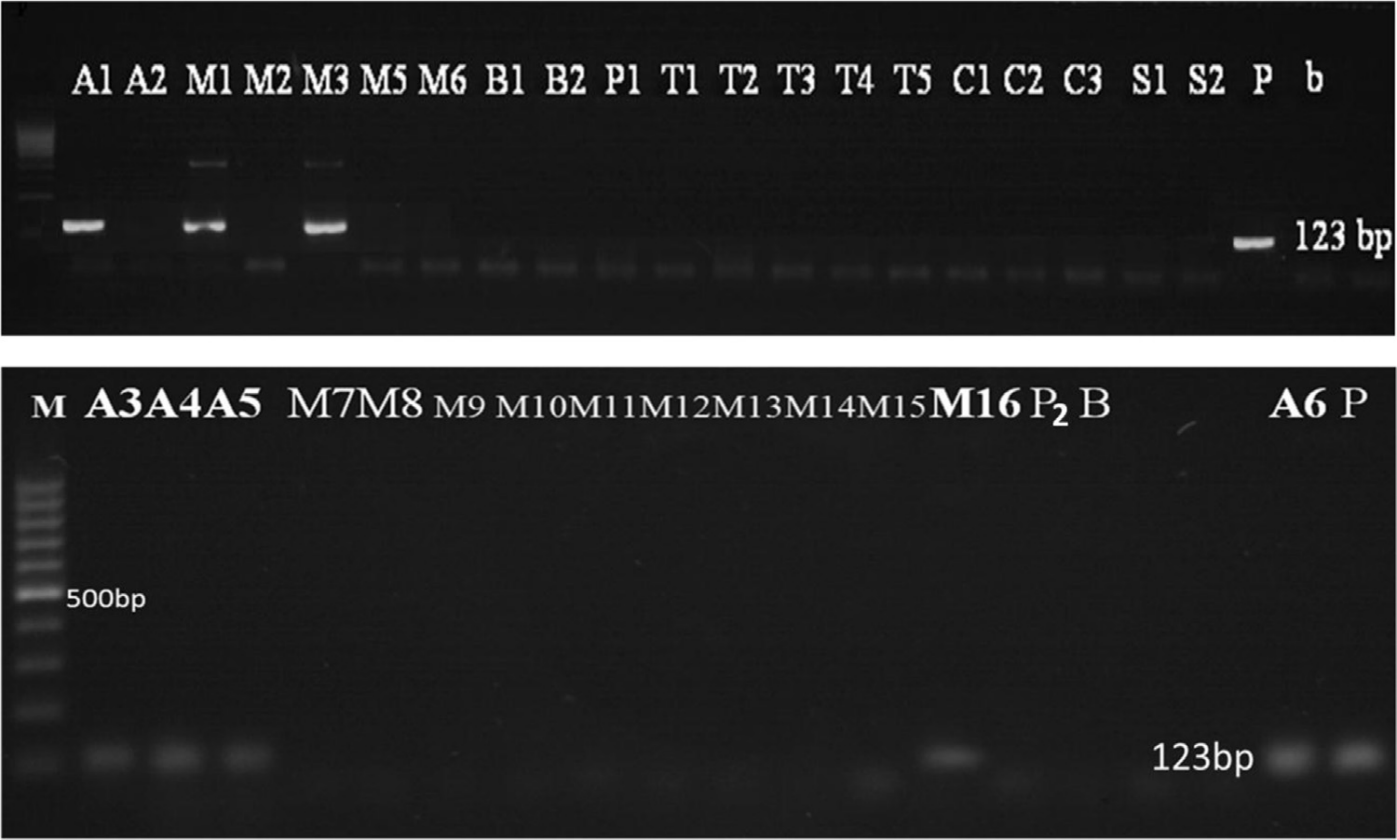
Detection of 35 S promoter in samples (Results of PCR products of primer pair p35S-cf3 and p35S-cf4), **M**: 100 bp DNA marker, **B**: Negative control, **P**: positive control plasmid (PGIIMH35-2PS), Lanes **A1–P2**, **A6**: Tested samples.

**Fig. 4:**
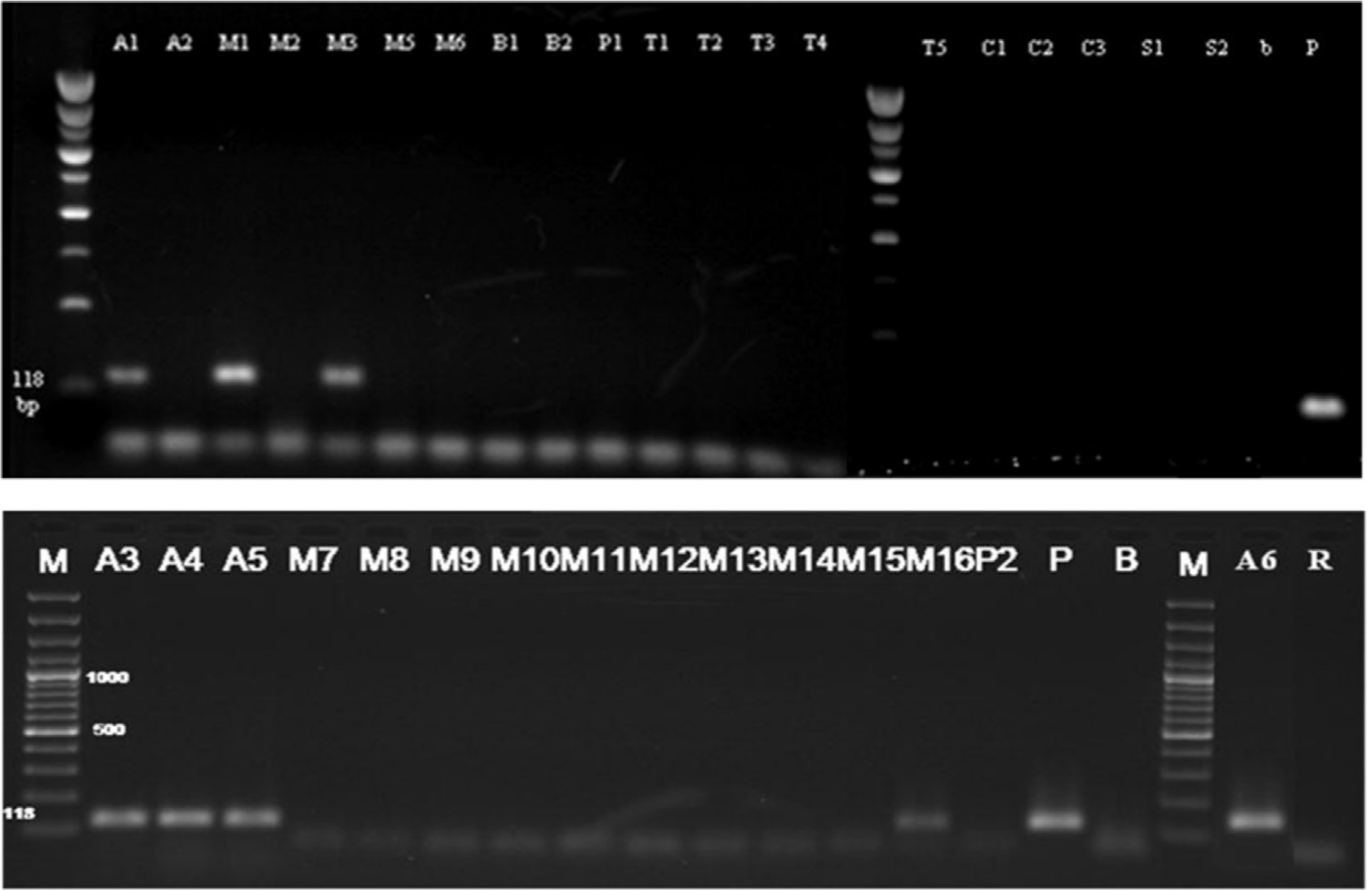
Detection of *nos* terminator in samples (Results of PCR products of primer pair HA-nos118-f /HA-nos118-r), **M**: 100 bp DNA marker, **B**: Negative control, **P**: positive control plasmid (PGIIMH35-2PS), Lanes **A1–P2**: Tested samples.

The authors would like to apologize for any inconvenience caused to the readers of the journal.

